# Poor perceived oral health is associated with adverse mental health outcomes among Syrian refugees in Canada

**DOI:** 10.1371/journal.pgph.0003824

**Published:** 2024-11-01

**Authors:** Jamil Alghanem, Salsabil Haque, Khansa Ababneh, Hana M. A. Fakhoury, Safoura Zangiabadi, Hala Tamim

**Affiliations:** 1 College of Medicine, Alfaisal University, Riyadh, Saudi Arabia; 2 Department of Preventive Dental Science, College of Dentistry, King Saud bin Abdulaziz University for Health Sciences, Riyadh, Saudi Arabia; 3 King Abdullah International Medical Research Center, Riyadh, Saudi Arabia; 4 Ministry of the National Guard ‐ Health Affairs, Riyadh, Saudi Arabia; 5 Department of Kinesiology and Health Science, York University, Toronto, Ontario, Canada; Keele University, UNITED KINGDOM OF GREAT BRITAIN AND NORTHERN IRELAND

## Abstract

While inadequate oral health has been linked to adverse mental health outcomes, there is limited understanding of such implications among refugees who bear a disproportionate burden of oral health disparities. This study aims to examine the effect of self-rated oral health on depression, anxiety, and stress among Syrian refugee parents resettled in Ontario. In this cross-sectional study, a total of 540 Syrian refugee parents who resided in Ontario for an average of 4 years and had at least one child under 18 years old were interviewed between March 2021 and March 2022. Information about self-rated oral health was gathered based on the question “In general, how would you rate the health of your teeth and mouth”. Responses ranged from 1 representing “excellent” and 5 representing “very poor”. The mean score (SD) of self-rated oral health was 3.2 (1.2). Mental health outcomes of depression, anxiety, and stress were measured using the Depression Anxiety Stress Scales (DASS-21). Multiple linear regression analyses were performed to assess the independent relationship between self-rated oral health and depression, anxiety, and stress, adjusting for other variables including, sociodemographic-, migration-, and health-related factors. Among participants, 6.3% rated their oral health as excellent, 26.9% as good, 23.1% as fair, 24.8% as poor, and 18.7% as very poor. Results of the multiple linear regression analyses indicated that poorer self-rated oral health was significantly associated with higher levels of depression (Adjβ = 0.98; p = 0.002; 95% CI = 0.38–1.59), anxiety (Adjβ = 1.03; p< 0.001; 95% CI = 0.54–1.52), and stress (Adjβ = 1.25; p< 0.001; 95% CI = 0.61–1.88). Further efforts and targeted interventions are needed to address the unmet oral health needs of Syrian refugees to improve mental health outcomes within this vulnerable population.

## Introduction

Oral health, which contributes to 18.3 billion years of healthy life lost or lived with disability (a measure known as ‘disability-adjusted life-years’) and an economic burden of 544 billion US dollars [1], remains a critical public health issue. Defined as the overall health of the mouth and encompassing conditions like tooth decay, gum disease, and oral cancers, poor oral health has been linked to numerous adverse physical and mental health outcomes [2–4]. Studies demonstrate associations with low cardiorespiratory fitness [4], endocarditis [5], pregnancy complications [6], and, critically, mental health concerns [7, 8]. The COVID-19 pandemic has further exacerbated these connections, particularly among vulnerable populations already facing healthcare disparities [7]. Existing research suggests a complex interplay between biological, social, and psychological factors in the oral-mental connection. For instance, a 2022 systematic review highlighted a potential link between oral health conditions and mental disorders, mediated by the oral microbiome, with circulating endotoxins being one possible mechanism [8]. Additionally, a 2016 study among individuals with major depressive disorder (MDD) hypothesized that inflammation originating from oral diseases might be another mechanism, potentially affecting neurotransmitter function and neurogenesis [9]. Furthermore, research has shown that people from lower socioeconomic strata bear a disproportionately high burden of dental caries and periodontal disease [10].

Among refugees, there is a significant burden of oral health problems [11, 12], which stems from limited access to dental care both before and after migration, challenges in integrating into the healthcare system, and pre-existing oral health issues from their home country [13]. Studies have also shown high rates of unmet dental needs and untreated dental caries among this population [12, 14]. This disparity translates to pain, discomfort, and reduced quality of life, highlighting the need for targeted interventions [15].

The ongoing Syrian civil war has displaced millions, with over 50,000 individuals now residing in Canada, nearly half of whom have resettled in Ontario [16]. Although programs like the Interim Federal Health Program (IFHP) provide temporary health coverage, its limitations leave many refugees facing access barriers [17]. Research suggests that Syrian refugees in Canada often have unmet healthcare needs, potentially linked to their sponsorship pathways and post-migration socioeconomic status [18]. Additional contributing factors include long wait times, high costs, and language barriers [14, 19]. Research in Nova Scotia reveal high rates of untreated tooth decay (85%) and gingivitis (98%) among refugees, alongside reported negative dental care experiences such as delayed consultations and limited treatment options [14]. Furthermore, Syrian refugees resettled in high-income Western countries experience a significantly higher prevalence of mental health disorders, including post-traumatic stress disorder (PTSD), depression, and anxiety, compared to the general population [20]. A systematic review conducted in 2020 suggests that syrian refugees are nearly ten times more likely to develop post-traumatic stress, depression and anxiety, highlighting the unique challenges they face [21]. For instance, a study referenced in Nguyen et al. [20] found that 29.5% of Syrian refugees resetlled in Jordan experienced symptoms of major depression, and 31.8% of Sryian refugees in Sweden exhibited anxiety.

Despite existing research on the oral health-mental health connection, no studies have specifically examined this relationship among Syrian refugees in Ontario. Understanding this link is crucial given the significant Syrian refugee population in this province and the need to ensure their overall health and well-being. This study aims to investigate the association between poor oral health and adverse mental health outcomes among Syrian refugees in Ontario. By exploring this relationship, we can inform targeted interventions, contribute to a holistic refugee healthcare framework, and advocate for appropriate funding and resources to address the specific health needs of this vulnerable population.

## Methodology

### Study participants

A total of 540 parents of Syrian refugees were recruited and interviewed for this cross-sectional study conducted between March 2021 and March 2022. Eligibility criteria included being a parent of a Syrian refugee child under the age of 18 at the time of the interview, residing in Ontario, Canada, and having resettled in the country after 2015. These criteria are based on the Government of Canada’s response to the Syrian refugee crisis and the resettlement plan initiated in 2015. Participants were recruited through convenience sampling with the assistance of community organizations like Access Alliance Multicultural Health and the Arab Community Centre of Toronto.

### Ethical considerations and data collection

The study was granted ethical approval by the York University Research Ethics Board (Certificate # e2019-128). To adhere to social distancing guidelines during the COVID-19 pandemic, the survey was conducted remotely via telephone. Prior to commencing the survey, the research assistants emailed a digital copy of the consent form to the participants. Moreover, participants were informed about the study’s objective and the voluntary nature of their involvement, with the option to opt out at any time without any consequences. The research assistant then thoroughly explained the consent form, addressing any questions or concerns raised by the participants. Subsequently, their oral consent was obtained for the record. Following informed consent, research assistants administered the questionnaire in Arabic. Participants’ responses were recorded using Qualtrics [22], a secure electronic data collection platform protected by a password. To compensate for their time and participation, individuals received a $20 honorarium. To ensure effective communication and adherence to COVID-19 safety protocols, the survey was administered remotely via telephone by research assistants fluent in Arabic, particularly the Syrian dialect.

### Demographic characteristics, self-rated oral health, and mental health assessment

Participants were assessed on various sociodemographic, migration, and health factors. These included gender role (Mother/Father), age, number of children, education level (None/Elementary/Secondary/High school diploma/University degree), perceived English/French proficiency (Scale from 1–6, where 1 = Excellent and 6 = Not at all proficient), working status (Employed/Unemployed), self-perceived socioeconomic status (Scale form 1–5, where 1 = Lower income and 5 = Upper income), sponsorship type (Governmental/Private/Other), years in Canada, alcohol and smoking habits, fear of COVID-19, and self-rated Physical Health (Scale from 1–5, where 1 = Excellent and 5 = Poor). Continuous variables were reported as mean (SD) and categorical variables as frequencies and percentages.

The main outcome of the study was mental health, specifically levels of depression, anxiety, and stress, among Syrian refugee parents in Ontario. For measuring mental health, we utilized the Depression, Anxiety, and Stress Scale (DASS-21) [23]. The DASS-21 is a widely recognized, self-reported tool containing 21 items, developed as a concise version of the original 42-item DASS [23]. It assesses the three aforementioned mental health domains based on experiences within the past week. Each subscale comprises seven items, using a 4-point Likert scale ranging from "Did not apply to me at all" to "Applied to me very much, or most of the time." Following the authors’ recommendations, we calculated total scores for each subscale by multiplying individual item scores by two, resulting in full-scale scores ranging from 0 to 42 [24]. These scores were then categorized into severity levels: normal, mild/moderate, and severe/extremely severe according to established cut-off points [24]. The Arabic translation of the 42-item DASS has demonstrated good Cronbach’s alpha reliability (0.93 for the depression subscale, 0.90 for the anxiety subscale, and 0.93 for the stress subscale) [25].

The independent variable of interest was self-rated oral health. This was assessed through the following question: "In general, how would you rate the health of your teeth and mouth?" Participants responded on a scale of 1 to 5, with 1 indicating "Excellent" and 5 indicating "Very Poor."

### Statistical analyses

Simple linear regression models were conducted to assess the bivariate relationship between self-rated oral health, sociodemographic-, migration-, and health-related factors with depression, anxiety, and stress. Additionally, three multiple linear regression models were fitted to explore the association between mental health outcomes (depression, anxiety, and stress) and self-rated oral health by adjusting other covariates such as sociodemographic-, migration-, and health-related variables. Multiple linear regression analyses were conducted after examining the assumptions and observing no significant violations. Moreover, clinically meaningful interactions were assessed to examine their potential impact on the relationships examined in the regression models and were found to be non-significant. The beta coefficient and 95% confidence intervals (95% CIs) were reported. All regression models were adjusted to account for the clustering effect arising from individuals belonging to the same family. All analyses were performed using the IBM SPSS v28.0 (Armonk, NY, USA).

## Results

### Characteristics of study participants and bivariate associations with mental health outcomes

**Table 1** presents the characteristics of the study participants. A total of 540 Syrian refugee parents residing in Ontario were recruited for this study. The mean age and number of children were 39.8 years (SD = 7.3) and 3.4 (SD = 1.5), respectively, with the majority of participants being mothers (60.9%). Among the participants, 81.3% had at least a high school education, while 65.6% were unemployed. Additionally, the average number of years spent in Canada since arrival was 3.90 years (SD = 1.6). Moreover, self-rated oral health ratings were distributed as followed: 6.3% excellent, 26.9% good, 23.1% fair, 24.8% poor, and 18.7% very poor.

**Table 1:** Characteristics of study participants.

Factors	N (%)	Mean (SD)
Self-rated Oral Health*		3.2 (1.2)
Socio-demographic		
**Gender**		
Mother	329 (60.9)	
Father	211 (39.1)	
**Age**		39.8 (7.3)
**Number of Children**		3.4 (1.5)
**Education**		
None/ Elementary	101 (18.7)	
Secondary/High school/ Diploma	278 (51.5)	
University	161 (29.8)	
Language proficiency**		3.0 (1.2)
**Working status**		
Yes	186 (34.4)	
No	354 (65.6)	
**Self-perceived socioeconomic status** ^**#**^		2.1 (1.0)
Migration		
**Sponsorship**		
Governmental	202 (37.4)	
Private	312 (57.8)	
Other	26 (4.8)	
**Number of years in Canada**		3.9 (1.6)
Health		
**Alcohol drinking**		
Yes	78 (14.4)	
No	462 (85.6)	
**Smoking**		
Yes	119 (22.0)	
No	421 (78.0)	
Self-rated Physical Health**		2.9 (1.1)
**Fear of COVID-19**		15.6 (6.0)

*Scale from 1–5 (1 = Excellent and 5 = Very poor)

**Scale from 1–6 (1 = Excellent and 6 = Not at all proficient)

^#^Scale form 1–5 (1 = Lower income and 5 = Upper income)

**Fig 1:**
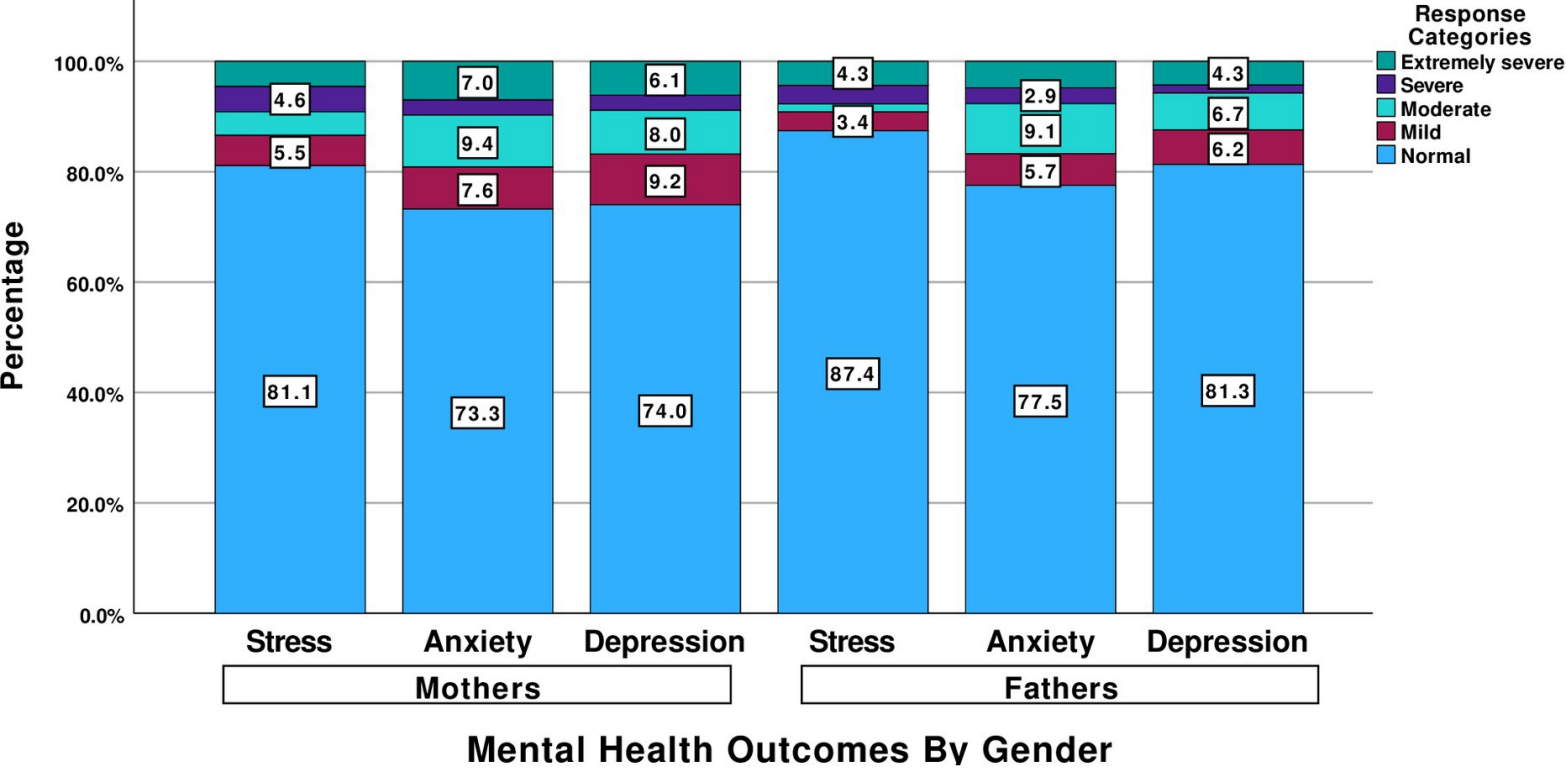
Mental health outcome of depression, anxiety, and stress among Syrian refugee parents in Canada.

Fig 1 demonstrates the levels of depression, anxiety, and stress among Syrian refugee mothers and fathers. The results illustrate that the majority of parents exhibited normal levels of depression, anxiety, and stress. Among mothers, 6.1%, 7.0% and 4.6% reported extremely severe levels of depression, anxiety and stress, respectively, compared to fathers who reported extremely severe levels at rates of 4.3%, 4.8%, and 4.3%, respectively.

### Multivariable analysis of mental health predictors

Table 2 presents the results of the bivariate and multiple linear regression analyses examining the associations between self-rated oral health, sociodemographic, migration, and health-related factors, and mental health outcomes. Bivariate regression analysis revealed a significant association between self-rated oral health and depression (Adjβ = -1.77; p < 0.001), anxiety (Adjβ = -1.68; p < 0.001), and stress (Adjβ = -2.03; p < 0.001). These findings indicate that participants with poorer self-rated oral health reported higher levels of depression, anxiety, and stress compared to those with better oral health. Results of the multiple linear regression models indicated overall R-squared values of 0.244, 0.310, and 0.286 for depression, anxiety, and stress, respectively. After adjusting for all other factors, individuals with poorer self-rated oral health exhibited significantly higher levels of depression (Adjβ = 0.98; p = 0.002; 95% CI: 0.38, 1.59), anxiety (Adjβ = 1.03; p = 0.001; 95% CI: 0.54, 1.52), and stress (Adjβ = 1.25; p = 0.001; 95% CI: 0.61, 1.88) compared to those with better self-rated oral health.

**Table 2:** Regression modelling of mental health outcomes in relation to self-rated oral health, sociodemographic, migration, and health-related factors.

Factors	Stress					Anxiety					Depression				
	Unadjusted β (SE)	p-value	Adjusted β (SE)	95%CI	p-value	Unadjusted β (SE)	p-value	Adjusted β (SE)	95%CI	p-value	Unadjusted β (SE)	p-value	Adjusted β (SE)	95%CI	p-value
Self-rated Oral Health*	2.03 (0.33)	**<0.001**	1.25 (0.32)	0.61, 1.88	**<0.001**	1.68 (0.26)	**<0.001**	1.03 (0.25)	0.54, 1.52	**<0.001**	1.77 (0.31)	**<0.001**	0.98 (0.31)	0.38, 1.59	**0.002**
Socio-demographic															
**Gender**															
Mother	ref					ref					ref				
Father	-1.89 (0.85)	**0.027**	-2.22 (0.94)	-4.06, -0.38	**0.018**	-1.24 (0.67)	0.064	-1.95 (0.72)	-3.36, -0.53	**0.007**	-1.25 (0.80)	0.117	-1.94 (0.90)	-3.71, -0.17	**0.031**
**Age**	0.003 (0.058)	0.961	-0.14 (0.06)	-0.25, -0.02	**0.026**	0.04 (0.05)	0.326	-0.07 (0.05)	-0.16, 0.02	0.148	0.05 (0.05)	0.365	-0.04 (0.06)	-0.16, 0.07	0.458
**Number of Children**	0.25 (0.29)	0.399	-0.34 (0.33)	-0.98, 0.31	0.302	0.41 (0.22)	0.067	-0.29 (0.25)	-0.78, 0.20	0.251	0.23 (0.27)	0.394	-0.06 (0.31)	-1.25, -0.03	**0.040**
**Education**															
None/ Elementary	ref					ref					ref				
Secondary/High school/ Diploma	-2.02 (1.13)	0.075	-1.00 (1.18)	-3.31, 1.31	0.396	-2.75 (0.88)	**0.002**	-1.27 (0.90)	-3.04, 0.51	0.161	-2.00 (1.05)	0.059	-0.70 (1.13)	-2.92, 1.52	0.537
University	-2.19 (1.26)	0.083	-0.08 (1.49)	-3.01, 2.85	0.957	-2.92 (0.98)	**0.003**	-0.14 (1.15)	-2.39, 2.12	0.905	-2.24 (1.17)	0.056	0.49 (1.43)	-2.33, 3.30	0.735
Language proficiency*	1.09 (0.34)	**0.001**	0.69 (0.42)	-0.13, 1.52	0.098	1.33 (0.26)	**< 0.001**	1.05 (0.32)	0.42, 1.69	**0.001**	1.17 (0.31)	**< 0.001**	0.78 (0.40)	-0.007, 1.57	0.052
**Working status**															
Yes	-1.34 (0.89)	0.132	1.55 (0.93)	-0.27, 3.36	0.096	-1.28 (0.70)	0.067	0.79 (0.71)	-0.61, 2.19	0.269	-1.78 (0.83)	**0.032**	-0.42 (0.89)	-1.33, 2.16	0.637
No	ref							ref			ref				
**Self-perceived SES** ^**#**^	-1.64 (0.41)	**<0.001**	-0.81 (0.39)	-1.57, -0.04	**0.039**	-1.22 (0.32)	**<0.001**	-0.45 (0.30)	-1.04, 0.14	0.136	-1.47 (0.38)	**<0.001**	-0.58 (0.37)	-1.31, 0.16	0.123
Migration															
**Sponsorship**															
Governmental	ref					ref					ref				
Private	-0.54 (0.89)	0.546	1.68 (0.97)	-0.22, 3.57	0.083	-0.55 (0.70)	0.436	1.57 (0.75)	0.11, 3.04	**0.035**	-1.56 (0.83)	0.062	-0.38 (0.93)	-2.20, 1.45	0.685
Other	1.99 (2.05)	0.333	1.95 (1.80)	-1.59, 5.49	0.280	2.63 (1.58)	0.097	2.11 (1.37)	-0.58, 4.80	0.124	0.61 (1.88)	0.745	-0.08 (1.71)	-3.43, 3.28	0.965
**Number of years in Canada**	0.24 (0.28)	0.393	0.31 (0.28)	-0.24, 0.85	0.266	0.32 (0.22)	0.151	0.56 (0.21)	0.14, 0.98	**0.009**	0.15 (0.26)	0.558	0.31 (0.27)	-0.22, 0.83	0.249
Health															
**Alcohol drinking**															
Yes	0.68 (1.20)	0.573	1.51 (1.13)	-0.71, 3.73	0.183	-0.69 (0.94)	0.462	1.05 (0.88)	-0.67, 2.77	0.232	-0.14 (1.12)	0.905	1.941 (1.09)	-0.20, 4.09	0.076
No	ref					ref					ref				
**Smoking**															
Yes	1.51 (1.01)	0.134	-0.04 (0.98)	-1.97, 1.89	0.967	2.35 (0.78)	**0.003**	1.26 (0.76)	-0.22, 2.75	0.096	1.97 (0.94)	0.036	0.89 (0.94)	-0.96, 2.74	0.346
No	ref					ref					ref		ref		
Self-rated Physical Health**	3.85 (0.35)	**< 0.001**	3.46 (0.37)	2.74, 4.18	**<0.001**	2.90 (0.27)	**< 0.001**	2.28 (0.28)	1.73, 2.84	**<0.001**	3.25 (0.33)	**< 0.001**	2.74 (0.35)	2.05, 3.43	**<0.001**
**Fear of COVID-19**	0.36 (0.07)	**< 0.001**	0.22 (0.06)	0.09, 0.34	**<0.001**	0.34 (0.05)	**< 0.001**	0.22 (0.05)	0.13, 0.32	**<0.001**	0.30 (0.06)	**< 0.001**	0.20 (0.06)	0.09, 0.32	**<0.001**

*Scale from 1–5 (1 = Excellent and 5 = Very poor)

**Scale from 1–6 (1 = Excellent and 6 = Not at all proficient)

^#^Scale form 1–5 (1 = Lower income and 5 = Upper income)

Furthermore, fathers reported significantly lower levels of depression (Adjβ = -1.94; p = 0.031; 95% CI: -3.71, -0.17), anxiety (Adjβ = -1.95; p = 0.007; 95% CI: -3.36, -0.53), and stress (Adjβ = -2.22; p = 0.018; 95% CI: -4.06, -0.38) compared to mothers. Notably, only individuals with poorer English or French language proficiency displayed significantly elevated levels of anxiety (Adjβ = 1.05; p = 0.001) compared to those with higher language proficiency.

Results also revealed a significant association between self-perceived socioeconomic status (SES) and stress levels (Adjβ = -0.81; p = 0.039; 95% CI: -1.57, -0.04), indicating that participants with lower self-perceived SES reported higher levels of stress. Additionally, poorer self-rated physical health was significantly associated with higher levels of depression (Adjβ = 2.74; p < 0.001; 95% CI: 2.05, 3.43), anxiety (Adjβ = 2.28; p < 0.001; 95% CI: 1.73, 2.84), and stress (Adjβ = 3.46; p < 0.001; 95% CI:2.74, 4.18), respectively. Similarly, greater fear of COVID-19 was significantly linked to higher levels of depression (Adjβ = 0.20; p < 0.001; 95% CI: 0.09, 0.32), anxiety (Adjβ = 0.22; p < 0.001; 95% CI: 0.13, 0.32), and stress (Adjβ = 0.22; p < 0.001; 95% CI: 0.09, 0.34).

## Discussion

This study investigated the association between self-reported oral health and depression, anxiety, and stress among Syrian refugee parents in Ontario. Our findings revealed a significant association, indicating that poorer perceived oral health was linked with higher levels of depression, anxiety, and stress. These results highlight the importance of addressing oral health issues as an integral component of comprehensive healthcare interventions and shed light on the substantial burden that poor oral health places on mental well-being.

A notable finding of our study was that 43.5% of Syrian refugee parents reported poor or very poor oral health, significantly exceeding the national average of less than 16% reported by the Canadian Dental Association [11, 26]. This also aligns with prior research documenting the disproportionately high burden of dental disease among refugee and immigrant populations in Canada compared to their native-born counterparts [27, 28]. Several factors contribute to this disparity. Pre-existing oral health problems from their home countries, documented in prior studies [29, 30], often go untreated due to limited access to dental care in Canada. Financial barriers, language difficulties, and unfamiliarity with the healthcare system can create a vicious cycle of neglect and worsening oral health [15]. Furthermore, emerging research suggests that stressors associated with resettlement can negatively impact mental health outcomes for refugees [31].

Our findings further demonstrated a significant association between poorer self-rated oral health and higher levels of depression, anxiety, and stress among Syrian refugee parents. This highlights the potential impact of oral health concerns on mental well-being within this vulnerable population. These results align with existing research suggesting a bidirectional relationship between oral and mental health [7, 32]. Similarly, another study reported that dental problems like tooth decay and tooth loss can lead to social isolation and reduced self-esteem, potentially contributing to poorer mental and overall health [7]. In the context of Syrian refugees, these findings are especially relevant due to the additional stressors and challenges they face [33]. Poor oral health might exacerbate existing mental health issues stemming from displacement, resettlement difficulties, and social isolation. Conversely, mental health struggles can also hinder oral health self-care. Conversely, mental health struggles can also hinder oral health self-care [7], creating a negative feedback loop.

Our findings align with emerging research exploring the potential biological mechanisms linking oral and mental health. A 2022 study investigated the role of oral microbiota in anxiety, mood, and trauma-related disorders. The study proposed that cytokines secreted by the host immune system in response to bacterial infection, potentially through mechanisms like neuroinflammation, might contribute to these mental health conditions [8]. Additionally, a 2016 study among individuals with major depressive disorder (MDD) explored the potential link between oral inflammation and treatment resistance [9]. The authors suggested that inflammation originating from oral diseases might affect neurotransmitter function and neurogenesis, hindering the effectiveness of MDD treatment [9]. While further research is needed, this proposes a biopsychosocial pathway through which poor oral health could impact mental well-being.

Similar to overall health, oral health is significantly impacted by social and environmental determinants [10]. Key social determinants include pre-existing health conditions, oral health literacy, behaviors, and access to dental services. Additionally, environmental factors, such as access to clean water and healthy food options, contribute to oral health outcomes [34, 35]. Moreover, the maintenance of oral health and treatment of its pathologies are influenced by government health policies and the extent of insurance coverage [36]. A scoping review published in March 2024 revealed that refugees are particularly affected by the social, environmental and political determinants of oral health mentioned above [37].

Beyond the association between oral health and mental health, our study identified other significant factors impacting mental health outcomes among Syrian refugee parents. Socioeconomic status (SES) emerged as a key factor, with individuals from lower SES experiencing higher levels of stress. This is consistent with prior studies, including a 2018 Danish research that reported a higher level of perceived stress among inhabitants of economically underprivileged regions [38]. This highlights the complex interplay between social determinants of health and mental well-being, particularly for refugee populations facing additional resettlement challenges and resource limitations. In this study, language proficiency was also linked to mental health, with participants reporting lower proficiency in English or French exhibiting higher levels of anxiety. This aligns with a 2019 US case study emphasizing the impact of language barriers and communication difficulties in healthcare settings [39]. For refugees navigating a new language and healthcare system, language proficiency can significantly impact access to care, social interaction, and overall well-being, potentially contributing to anxiety. Regarding gender, our findings align with previous research suggesting that women often experience higher levels of depression and anxiety compared to men [40]. This might be due to various factors, including societal expectations and gender roles. This disparity could also be linked to gendered social expectations, where women face greater pressure to maintain certain aesthetic standards. This heightened emphasis may increase oral health awareness (oral literacy), but limited access to dental care could potentially lead to increased psychological distress. Physical health also emerged as a significant predictor of mental health in our study, consistent with established research highlighting the bidirectional relationship between these domains [41]. Chronic physical conditions can contribute to stress, anxiety, and depression, while mental health struggles can negatively impact physical health behaviors and adherence to treatment plans. Finally, fear of COVID-19 was significantly associated with mental health outcomes in our study, reflecting the global anxiety and uncertainty surrounding the pandemic. This aligns with earlier research highlighting the psychological impact of COVID-19 on Syrian refugees facing additional stressors and vulnerabilities [42]. It is important to acknowledge that these associations are likely complex and influenced by various interacting factors. Future research exploring the specific mechanisms underlying these relationships, particularly within the context of Syrian refugee populations, is crucial.

While this study offers valuable insights into the relationship between self-rated oral health and mental health among Syrian refugee parents in Ontario, we acknowledge certain limitations. Firstly, the cross-sectional design prevents establishing causal relationships. It is possible that individuals with poorer mental health might neglect their oral health, leading to a perceived association in the opposite direction. Future longitudinal studies are needed to explore the temporal nature of this relationship. Secondly, participants’ subjective assessments of their oral health might be influenced by personal biases. Utilizing objective oral health examinations in future studies could mitigate this limitation. Thirdly, our data lacked information on participants’ oral health practices before immigration and their pre-existing mental health conditions. This could potentially introduce confounding bias, as factors influencing their oral health or mental health prior to resettlement might have remained unaddressed. Future studies could benefit from collecting a more comprehensive history of their health prior to immigration. Lastly, there is lack of detailed information on specific types of oral health issues among participants. Due to the study’s scope, such information regarding the oral health issues experienced by participants were not collected. Future research could benefit from conducting more detailed inquiries into the types and severity of oral health issues experienced by the participants to allow for a deeper understanding of the factors influencing self-rated oral health perceptions. Despite these limitations, our study contributes valuable knowledge to the growing body of research on the interconnectedness of oral and mental health. Particularly within the context of Syrian refugee populations facing unique challenges, recognizing and addressing oral health concerns holds significant potential for improving their overall well-being.

This study explored the association between self-rated oral health and mental health outcomes among Syrian refugee parents in Ontario. Our findings revealed a significant association, suggesting that poorer perceived oral health was linked with higher levels of depression, anxiety, and stress. These results highlight the potential interconnectedness of oral and mental health, particularly within vulnerable refugee populations. This study contributes to the growing body of evidence supporting the importance of integrating oral healthcare into comprehensive refugee health interventions. Addressing oral health concerns alongside mental health needs can potentially improve overall well-being and quality of life for refugees navigating resettlement challenges. However, further research is crucial to elucidate the underlying mechanisms linking oral and mental health. Additionally, identifying and evaluating effective interventions tailored to address both oral health and mental health needs of Syrian refugees is crucial. This could include culturally sensitive programs that promote oral health awareness, improve access to dental care, and address psychosocial factors impacting oral health behaviors. By prioritizing integrated approaches to oral and mental health care, we can better support the well-being and resilience of Syrian refugees in Canada.
